# Optimization of Extraction of Total Terpenoids from *Phellinus igniarius* Using Response Surface Methodology and Evaluation of Their Antioxidant Activity

**DOI:** 10.3390/molecules31111929

**Published:** 2026-06-03

**Authors:** Lulu Li, Zhe Wang, Jinbiao Hao, Ying Shao, Zhilong Qu, Zhenjiang Zhang, Anhui Chen, Zaizhong Ni, Yanan Wang

**Affiliations:** 1 College of Food and Bioengineering, Xuzhou University of Technology, Xuzhou 221018, China; luna310@xzit.edu.cn (L.L.);; 2 Anhui Yanhuang Biotechnology Co., Ltd., Hefei 230000, China

**Keywords:** *Phellinus igniarius*, total terpenoids, response surface methodology, extraction optimization, antioxidant activity

## Abstract

*Phellinus igniarius* is a medicinal fungus rich in terpenoids with potential biological activities. In this study, the *P. igniarius* terpenoid-enriched extract (PITT) was optimized using single-factor experiments combined with response surface methodology based on a Box–Behnken design. The optimal extraction conditions were determined as extraction temperature 64 °C, extraction time 21 min, ethanol concentration 78%, and liquid-to-solid ratio 20 mL/g, yielding a total terpenoid content of 7.078 mg/g. The chemical profile of PITT was characterized by liquid chromatography–tandem mass spectrometry (LC–MS/MS), revealing the presence of multiple putative terpenoid compounds, including betulin, curdione, and several sesquiterpenoids. The antioxidant activity of PITT was evaluated using DPPH, ABTS, and hydroxyl radical scavenging assays, showing dose-dependent effects. Furthermore, an H_2_O_2_-induced oxidative stress model in RAW 264.7 cells was employed to assess cellular antioxidant activity. Pretreatment with PITT significantly reduced intracellular reactive oxygen species (ROS) levels, enhanced the activities of endogenous antioxidant enzymes (superoxide dismutase, catalase, and glutathione peroxidase), and decreased malondialdehyde (MDA) levels. These results indicate that PITT exhibits notable antioxidant activity and may serve as a potential natural antioxidant source for further development. However, further studies are required to elucidate the underlying mechanisms.

## 1. Introduction

Oxidative stress is implicated in the onset and progression of chronic diseases, including cancer, neurodegenerative disorders, and cardiovascular diseases [1,2,3]. Consequently, substantial effort has been devoted to identifying effective natural antioxidants from edible and medicinal organisms with a long history of human use.

Among medicinal macrofungi, *Phellinus igniarius* (Polyporaceae, Basidiomycota) has been widely used in East Asia, including Traditional Chinese Medicine, Korean Medicine, and Japanese Kampo. It commonly grows on dead or fallen trunks of broad-leaved trees, such as *Populus* and *Betula* [4]. In traditional practice, *P. igniarius* has been used for “clearing heat and detoxifying”, promoting blood circulation, removing blood stasis, and strengthening the body [5,6]. In recent years, *P. igniarius* has attracted increasing attention as a dual-use resource aligned with the concept of “medicine–food homology”. Phytochemical investigations have revealed diverse bioactive constituents, including terpenoids, polysaccharides [7], and flavonoids, which have been associated with anti-inflammatory, antioxidant, and antitumor activities [8,9,10,11].

Terpenoids in medicinal fungi may contribute to antioxidant defense through multiple mechanisms, including direct radical scavenging, metal-ion chelation, and modulation of endogenous antioxidant enzymes [12,13]. Consistently, terpenoid-rich extracts from *P. igniarius* have demonstrated radical-scavenging activities [5,9,14]. However, comprehensive compositional profiling and systematic evaluation of how specific terpenoid subclasses contribute to antioxidant capacity remain limited, and the structure-activity relationships underlying these effects are not well established.

Efficient isolation of terpenoids from *P. igniarius* remains a bottleneck, as conventional solvent extraction often requires prolonged processing and offers limited efficiency. Although antioxidant activities of *P. igniarius* extracts have been reported [15,16], the extent to which terpenoids account for these effects remains unclear. In this study, key parameters for extracting total terpenoids from the fruiting bodies of *P. igniarius* were optimized using response surface methodology (RSM). The resulting *P. igniarius* terpenoid-enriched extract (PITT) were characterized by LC-MS/MS and evaluated for antioxidant capacity using multiple in vitro assays and an H_2_O_2_-induced oxidative injury model in RAW 264.7 cells. This work provides an optimized strategy for obtaining antioxidant terpenoid fractions from *P. igniarius* and supports their further development for functional food and related applications.

## 2. Results and Discussion

### 2.1. Optimization of Extraction Process for PITT

#### 2.1.1. Single-Factor Effects of Liquid-to-Solid Ratio

**Figure 1:**
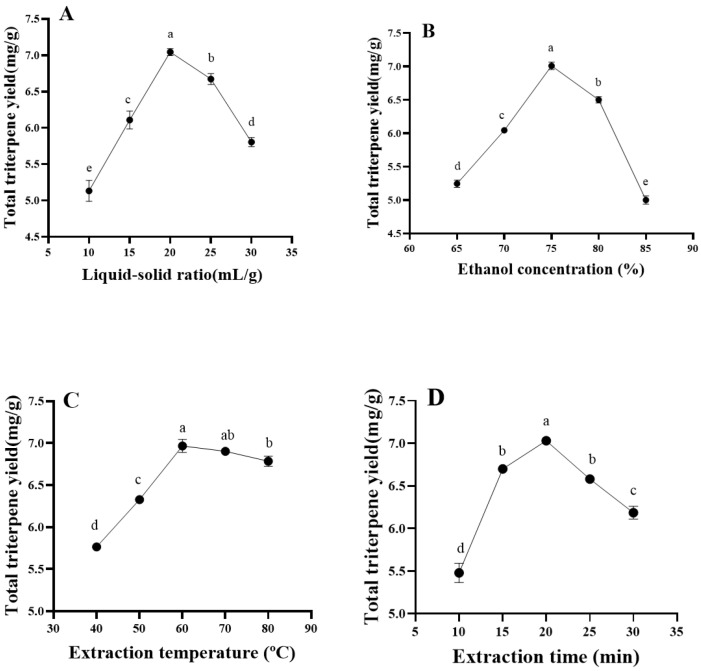
Effect of liquid-solid ratio (**A**), ethanol concentration (**B**), extraction temperature (**C**), and extraction time (**D**) on PITT yield. Data are presented as mean ± standard deviation (SD) of triplicate measurements. Different letters indicate significant differences among groups (*p* < 0.05).

Appropriate solvent volume is essential for efficient extraction of bioactive compounds from fungi. An insufficient solvent volume can limit the mass transfer of target compounds, whereas an excessive solvent volume increases solvent consumption and waste generation [17]. Similar trends have been reported for triterpene extraction from *Ganoderma lucidum* [18] and *Sanghuangporus sanghuang* [5]. In the present study, the total terpenoid yield from *P. igniarius* was evaluated at liquid-to-solid ratios of 10, 15, 20, 25, and 30 mL/g (Figure 1A). The yield increased gradually from 5.13 to 7.04 mg/g as the ratio increased from 10 to 20 mL/g, but decreased at 30 mL/g. Therefore, a liquid-to-solid ratio of 20 mL/g was selected for subsequent experiments.

#### 2.1.2. Single-Factor Effects of Ethanol Concentration

Ethanol was selected as the extraction solvent for several reasons. First, ethanol–water mixtures provide tunable polarity that can be optimized for terpenoid extraction; terpenoids range from non-polar (e.g., camphor) to moderately polar (e.g., abscisic acid), and ethanol concentrations between 60–80% have been shown to maximize triterpene recovery from medicinal fungi [5,18]. Second, ethanol is Generally Recognized as Safe (GRAS) for food-related applications, making it suitable for functional food research. Third, compared to methanol or chloroform, ethanol offers a better balance between extraction efficiency and safety for downstream bioactivity assays. The polarity of 75% ethanol (dielectric constant ~45) is well-suited for extracting oxygenated terpenoids while limiting co-extraction of non-polar lipids. To identify an appropriate ethanol concentration, total terpenoid extraction was assessed at 65%, 70%, 75%, 80%, and 85% ethanol (Figure 1B). The yield increased as ethanol concentration rose from 65% to 75% and then decreased when ethanol concentration further increased from 75% to 85%. The highest PITT yield (7.01 ± 0.045 mg/g) was obtained at 75% ethanol, and differences among groups were significant (*p* < 0.05). Similar observations have been reported in other triterpene extraction studies [5]. A moderate ethanol proportion (75%) may provide an optimal solvent polarity for terpenoids, balancing solubility and selective extraction; however, further increases in ethanol content may co-extract more non-target ethanol-soluble components without improving terpenoid recovery, thereby reducing apparent extraction efficiency [19]. Accordingly, 75% ethanol was selected for subsequent experiments.

#### 2.1.3. Single-Factor Effects of Extraction Temperature

Extraction temperature can influence extraction efficiency by affecting solvent viscosity, solute diffusivity, and solubility. Temperatures of 40, 50, 60, 70, and 80 °C were evaluated (Figure 1C). The total terpenoid yield increased as temperature increased from 40 to 60 °C, consistent with enhanced mass transfer at elevated temperatures [17]. When temperature exceeded 60 °C, the yield decreased slightly, possibly due to thermal degradation of heat-sensitive terpenoids. Therefore, 60 °C was selected as the optimal extraction temperature.

#### 2.1.4. Single-Factor Effects of Extraction Time

Extraction time is another important factor affecting recovery of target compounds. Extraction times of 10, 15, 20, 25, and 30 min were evaluated (Figure 1D). The total terpenoid yield increased from 10 to 20 min and reached a maximum at 20 min, after which it decreased with further extension of extraction time. The plateau at 20 min reflects complete mass transfer of accessible terpenoids; prolonged extraction may promote degradation of thermolabile terpenoid structures or co-extract competing chromophores [19]. Consequently, 20 min was selected as the optimal extraction time.

#### 2.1.5. RSM-Based Optimization of the Process for Total Terpenoids Extraction

In experimental design, statistical methods are widely used to model and analyze systems in which multiple factors influence the response. Response surface methodology (RSM) is an efficient optimization framework that integrates statistical and mathematical techniques to develop empirical models and explore factor-response relationships [18]. The Box–Behnken design (BBD) is commonly used in RSM because it efficiently estimates quadratic models while avoiding experimental runs in which all factors are simultaneously set at their extreme levels. Five center points were chosen to provide an independent estimate of pure error and to assess model lack-of-fit, which is critical for validating response surface curvature. In a BBD, each factor is studied at three levels, and the design is suitable for fitting second-order (quadratic) models; therefore, it has been widely adopted in RSM studies [20].

A quadratic polynomial model was fitted to the experimental data (Table S3) using Design-Expert software, yielding the following regression equation:

Y = 7.01 − 6.625 × 10^−3^A + 0.34B + 0.32C − 5.000 × 10^−3^AB + 3.250 × 10^−3^AC + 0.017BC − 0.48A^2^ − 0.49B^2^ − 0.89C^2^, (1)

where Y is the total terpenoid yield (mg/g), A is extraction time (min), B is extraction temperature (°C), and C is ethanol concentration (%). The model showed a good fit to the experimental data (R^2^ = 0.9964).

ANOVA results (Table 1) indicated that the model was highly significant (*p* < 0.0001), while the lack-of-fit was not (*p* = 0.5908). Among the linear terms, extraction temperature (B) and ethanol concentration (C) had significant effects on yield (*p* < 0.0001), whereas extraction time (A) did not (*p* = 0.7746). All quadratic terms (A^2^, B^2^, C^2^) were highly significant (*p* < 0.0001), with C^2^ showing the strongest curvature effect. None of the interaction terms (AB, AC, BC) reached statistical significance (*p* > 0.05).

**Figure 2:**
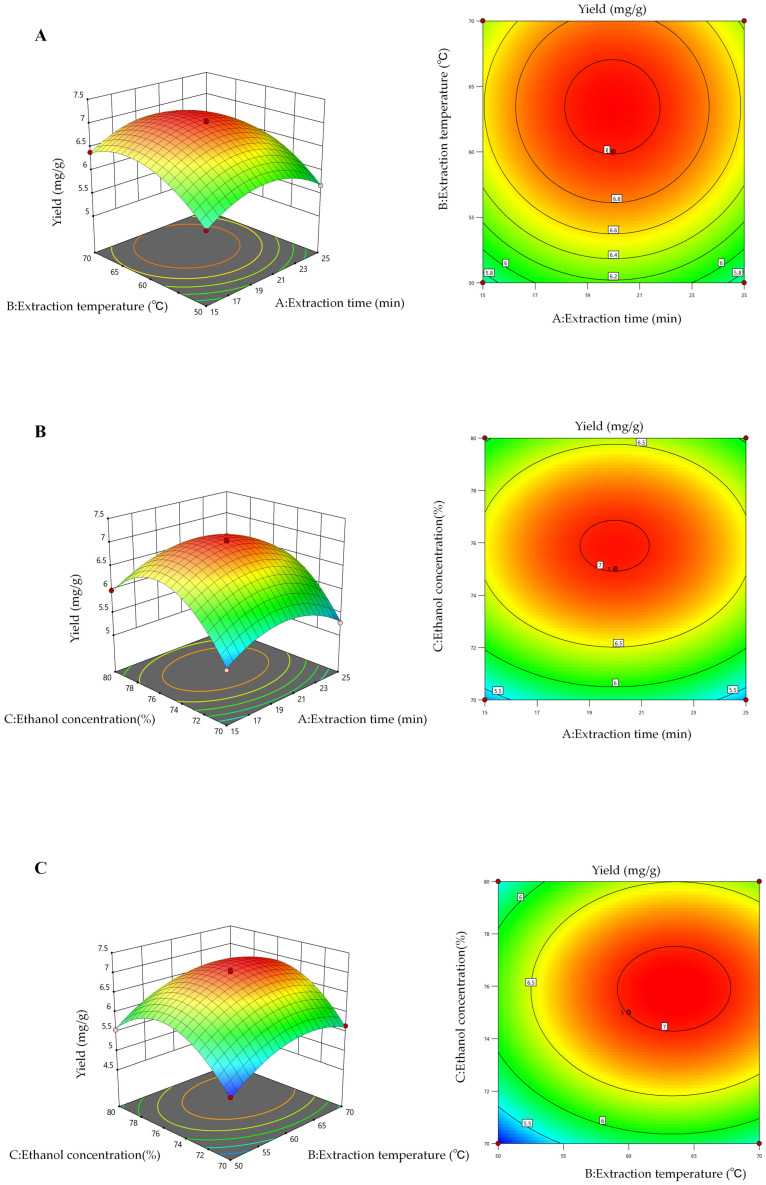
Response surface 3D diagram and contour diagram of the interactions among the three variables affecting the PITT yield. (**A**) Extraction time and extraction temperature. (**B**) Extraction time and ethanol concentration. (**C**) Extraction temperature and ethanol concentration. Colors from red to blue represent higher to lower predicted total terpenoid yields, respectively.

Both two-dimensional (2D) contour plots and three-dimensional (3D) response surface plots, derived from the fitted regression model, can be used to visualize the relationships between the response and the independent variables [21]. The effects of extraction time (A), extraction temperature (B), and ethanol concentration (C) on the PITT yield were evaluated at a fixed liquid-to-solid ratio of 20 mL/g (Figure 2). The response surface plots showed convex shapes with a distinct maximum within the design space, confirming that the selected factor ranges were appropriate. Solving the regression equation gave the predicted optimal conditions: temperature 63.47 °C, ethanol 77.22%, and time 20.39 min, with a predicted yield of 7.078 mg/g. Confirmatory experiments under practical conditions (64 °C, 78% ethanol, 21 min) yielded 7.078 ± 0.061 mg/g, validating the model. Of note, the total terpenoid yield represented less than 1% of the crude extract mass, suggesting the presence of substantial co-extracted non-terpenoid compounds (likely polysaccharides and phenolics). LC-MS/MS profiling confirmed non-terpenoid signals, indicating that the crude extract is compositionally complex. Future studies should characterize these components to fully understand their contribution to bioactivity.

### 2.2. Identification of PITT Through LC-MS/MS

Terpenoids are among the major bioactive constituents of *P. igniarius* [22]. In the present study, terpenoids were extracted from *P. igniarius* and further enriched for compositional analysis. Because terpenoids in *P. igniarius* are structurally diverse and occur in a complex matrix, their separation and identification remain challenging. Therefore, the terpenoid profile of PITT was characterized by LC-MS/MS. The total ion chromatograms (TICs) are shown in Figure S2 and representative MS/MS spectra for each compound are provided in Supplementary Table S4. The major terpenoids tentatively identified are summarized in Table 2, along with their previously reported biological activities (e.g., antioxidant, anti-inflammatory, and antitumor properties), which support the traditional use of *P. igniarius* in medicinal applications. All identifications are considered tentative and should be confirmed by authentic standards in future studies.

Curdione, a prominent sesquiterpene identified in this study, has been reported to exhibit a range of biological activities. Previous studies indicate that curdione possesses antitumor and anti-inflammatory properties, potentially involving the inhibition of platelet aggregation and modulation of apoptosis-related pathways in tumor cells [23,24]. In addition, its antioxidant capacity may contribute to the scavenging of free radicals and protection against oxidative damage.

Another major compound identified in PITT was atractylenolide II, which has been reported to inhibit proliferation in several cancer cell lines [31]. Mechanistic studies suggest that atractylenolide II can induce apoptosis and cause cell-cycle arrest, highlighting its potential relevance to anticancer research [30]. Its anti-inflammatory activity may also contribute to the medicinal effects attributed to *P. igniarius* extracts [50].

In addition to these compounds, several oxygenated terpenoid-like structures were annotated, including 3,7,15-trihydroxy-12,13-epoxytrichothec-9-en-8-one and 4-hydroxy-4a,8-dimethyl-3-methylene-3,3a,4,4a,7a,8,9,9a-octahydroazuleno[6,5-b]furan-2,5-dione. The detection of these highly functionalized sesquiterpenoids in PITT expands the chemical diversity of terpenoids reported from P. igniarius and provides a basis for future investigation of their potential bioactivities.

Overall, LC-MS/MS profiling revealed that PITT contains a chemically diverse set of terpenoids. The presence of these bioactive molecules-supported by published evidence of antitumor, antioxidant, and anti-inflammatory activities-highlights the potential for further investigation of *P. igniarius* as a natural source of bioactive compounds with reported pharmacological activities. Identification of these specific constituents provides a foundation for further mechanistic investigations and the development of PITT-based functional applications.

Future studies should characterize the non-terpenoid fraction (particularly phenolics) to dissect their contribution to the observed antioxidant activity.

### 2.3. In Vitro Antioxidant Capacity of PITT

Natural antioxidants, including terpenoids, are widely distributed in fungi [51]. Given the terpenoid-enriched nature of PITT, its antioxidant potential was evaluated using multiple in vitro assays.

**Figure 3:**
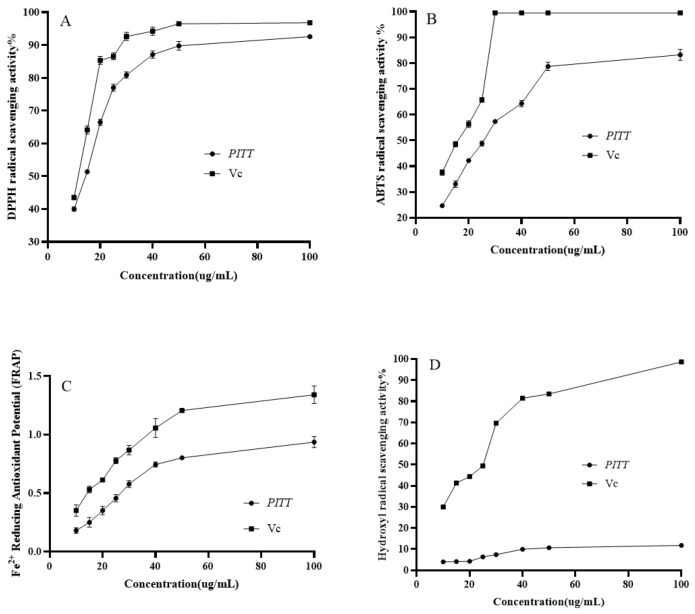
In vitro antioxidant activity of PITT. (**A**) DPPH radical scavenging activity, (**B**) ABTS radical scavenging activity, (**C**) ferric reducing antioxidant power (FRAP), and (**D**) hydroxyl radical scavenging activity. Vc was used as a positive control. Data are presented as mean ± standard deviation (SD) of triplicate measurements.

As shown in Figure 3, PITT exhibited concentration-dependent antioxidant activity. In the DPPH assay (Figure 3A), the scavenging rate increased from 39.9 ± 0.54% at 10 µg/mL to 92.21 ± 0.82% at 100 µg/mL, approaching the activity of vitamin C (Vc) at higher concentrations, although Vc reached a plateau earlier. The half-maximal inhibitory concentration (IC_50_) of PITT in the DPPH assay was determined to be 18.65 µg/mL. In the ABTS assay (Figure 3B), PITT also showed a clear concentration-dependent increase (24.68 ± 0.22% to 83.26 ± 0.22% from 10 to 100 µg/mL), with an IC_50_ value of 27.56 µg/mL, but remained lower than Vc across the tested range. For hydroxyl radicals (Figure 3D), PITT exhibited comparatively weak scavenging activity (4.01 ± 0.33% to 11.8 ± 0.56% from 10 to 100 µg/mL), indicating limited effectiveness against ·OH under the present conditions.

In addition, the ferric reducing antioxidant power (FRAP) assay was used to assess the reducing capacity of PITT. The reducing power increased with increasing concentration (200–1000 µg/mL; Figure 3C), but remained lower than that of Vc, suggesting that PITT has measurable electron-donating ability, albeit weaker than the positive control.

The non-linear concentration–response relationship, particularly the plateau at higher concentrations in DPPH and ABTS assays, may result from (i) saturation of radical species available for scavenging, (ii) self-aggregation of terpenoid molecules at high concentrations reducing their accessibility, or (iii) competitive inhibition among multiple terpenoid species with different radical-scavenging kinetics.

### 2.4. PITT Attenuates H_2_O_2_-Induced Oxidative Injury in RAW 264.7 Cells

#### 2.4.1. Effect of PITT on RAW264.7 Cell Viability

**Figure 4:**
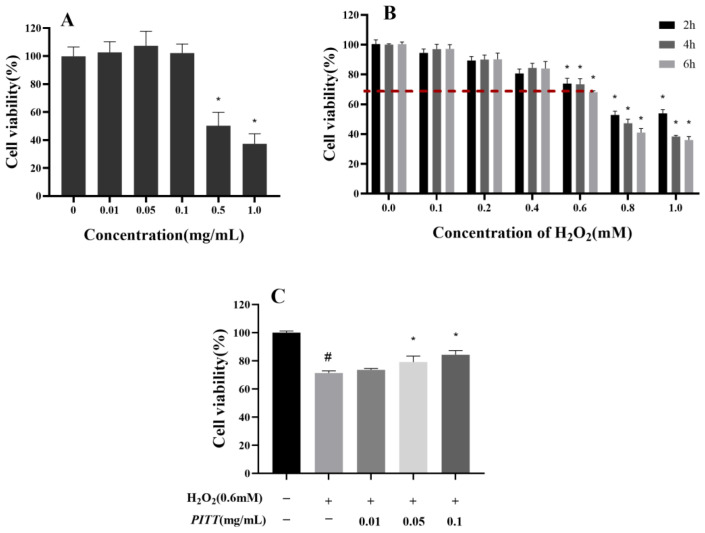
Effects of PITT on RAW 264.7 cell viability. (**A**) Cell viability after PITT treatment (0.01–1.0 mg/mL) for 24 h measured by the CCK-8 assay. (**B**) Cell viability after H_2_O_2_ treatment (0–1.0 mM) for 2, 4, or 6 h. The red line indicates the selected oxidative injury condition (0.6 mM H_2_O_2_, 6 h) with cell viability of approximately 68%. (**C**) Protective effect of PITT pretreatment (0.01–0.1 mg/mL) against H_2_O_2_-induced oxidative injury (0.6 mM) in RAW 264.7 cells, assessed by the CCK-8 assay. Data are presented as mean ± SD (*n* = 3). * *p* < 0.05 versus control group in figure (**A**,**B**); ^#^ *p* < 0.05 versus control group; * *p* < 0.05 versus oxidative damage group in figure (**C**).

To evaluate the antioxidant potential of PITT at the cellular level, its cytotoxicity toward RAW 264.7 cells was first assessed. As shown in Figure 4A, PITT at 0.01–0.1 mg/mL did not significantly affect cell viability compared with the control group (* *p* > 0.05). In contrast, cell viability decreased significantly at 0.5 and 1.0 mg/mL (* *p* < 0.05). Therefore, PITT concentrations of 0.01–0.1 mg/mL were selected for subsequent experiments.

#### 2.4.2. Effect of H_2_O_2_ on RAW264.7 Cell Viability

Hydrogen peroxide (H_2_O_2_) is commonly used to induce oxidative stress in vitro [52] and can disrupt cellular metabolism, including fatty acid and amino acid pathways [53]. To establish an oxidative injury model, RAW 264.7 cells were treated with different H_2_O_2_ concentrations for 2, 4, or 6 h, and cell viability was assessed (Figure 4B). H_2_O_2_ at 0.6–1.0 mM significantly reduced cell viability (* *p* < 0.05). Notably, treatment with 0.6 mM H_2_O_2_ for 6 h resulted in a viability of 68.11 ± 0.91% relative to the untreated control, which falls within the commonly accepted range (50–70%) for a moderate and reproducible injury model. Accordingly, 0.6 mM H_2_O_2_ for 6 h was selected for subsequent experiments.

#### 2.4.3. Protective Effect of PITT Against H_2_O_2_-Induced Oxidative Injury in RAW 264.7 Cells

The H_2_O_2_-induced oxidative injury model in RAW 264.7 cells is widely used to evaluate antioxidant protection in vitro [54]. As shown in Figure 4C, H_2_O_2_ exposure markedly decreased cell viability compared with the control group (# *p* < 0.05). Pretreatment with PITT improved cell viability in a concentration-dependent manner. In particular, 0.05 and 0.1 mg/mL PITT significantly increased cell viability compared with the oxidative damage group (* *p* < 0.05), whereas 0.01 mg/mL showed a slight but non-significant improvement. These results indicate that PITT can attenuate H_2_O_2_-induced oxidative injury in RAW 264.7 cells within the non-cytotoxic concentration range.

#### 2.4.4. ROS Assay in H_2_O_2_-Induced RAW 264.7 Cells

Intracellular ROS accumulation is a key event in H_2_O_2_-induced oxidative injury [55]. Therefore, the effect of PITT on intracellular ROS levels was evaluated using the fluorescent probe DCFH-DA. DCFH-DA is cell-permeable and nonfluorescent; once inside cells, it is deacetylated to DCFH and subsequently oxidized by ROS to form the fluorescent product DCF, which emits green fluorescence [56]. Accordingly, DCF fluorescence intensity was used as an indicator of intracellular ROS levels.

**Figure 5:**
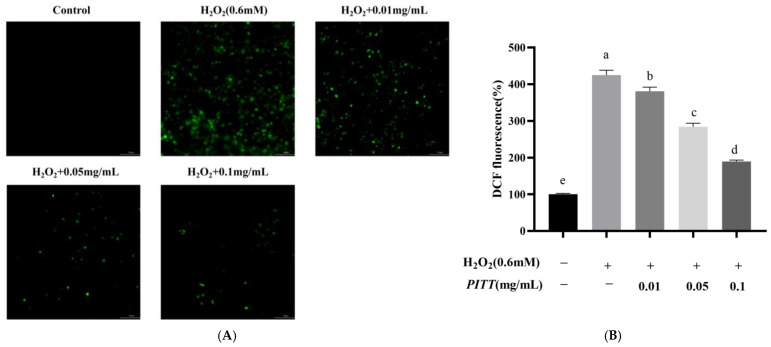
Effects of PITT on H_2_O_2_-induced ROS production in RAW 264.7 cells. (**A**) Representative fluorescence images of DCF staining in RAW 264.7 cells under different treatments. (**B**) Quantification of DCF fluorescence intensity. Cells were stained with DCFH-DA and observed by fluorescence microscopy. Data are presented as mean ± SD (*n* = 3). Different letters indicate significant differences among groups (*p* < 0.05, one-way ANOVA with Tukey’s post hoc test). Scale bar = 100 μm.

As shown in Figure 5A,B, exposure to 0.6 mM H_2_O_2_ markedly increased DCF fluorescence compared with the control group, indicating elevated intracellular ROS. Pretreatment with PITT (0.01–0.1 mg/mL) reduced H_2_O_2_-induced ROS accumulation in a concentration-dependent manner, as evidenced by the progressive decrease in fluorescence intensity. Quantitative analysis (Figure 5B) further confirmed significant differences among groups (different letters, *p* < 0.05), supporting the inhibitory effect of PITT on ROS generation.

#### 2.4.5. Cellular Antioxidant Capacity Under H_2_O_2_ Treatment

Endogenous antioxidant enzymes, including superoxide dismutase (SOD), catalase (CAT), and glutathione peroxidase (GSH-Px), constitute a primary defense system against oxidative stress by detoxifying reactive oxygen species (ROS) and their derived peroxides [57]. Malondialdehyde (MDA), a major end-product of lipid peroxidation, is commonly used as an indicator of oxidative damage to cellular membranes. Reduced glutathione (GSH) is a key non-enzymatic antioxidant that participates in redox homeostasis and protects cells against oxidative injury [58]. To further evaluate the protective effect of PITT, antioxidant enzyme activities (SOD, CAT, and GSH-Px), GSH content, and MDA levels were measured in RAW 264.7 cells.

**Figure 6:**
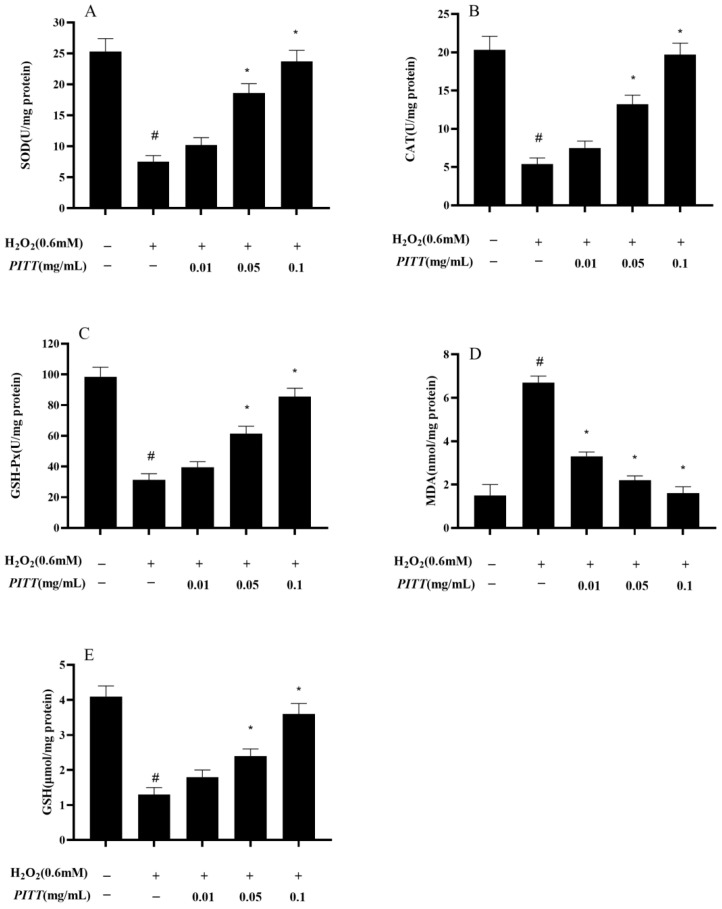
Effects of PITT on cellular antioxidant status in H_2_O_2_-induced RAW 264.7 cells. (**A**) SOD, (**B**) CAT, (**C**) GSH-Px, (**D**) MDA, and (**E**) GSH. Data are presented as mean ± SD (*n* = 3). # *p* < 0.05 versus the control group; * *p* < 0.05 versus the oxidative damage (H_2_O_2_) group.

As shown in Figure 6A–C,E, exposure to 0.6 mM H_2_O_2_ significantly decreased the activities/levels of SOD, CAT, and GSH-Px, as well as GSH content, compared with the control group (# *p* < 0.05). Pretreatment with PITT mitigated these decreases in a concentration-dependent manner. In particular, PITT at 0.05 and 0.1 mg/mL markedly restored antioxidant enzyme activities and GSH levels relative to the oxidative damage group (* *p* < 0.05), with the 0.1 mg/mL group showing values approaching the control levels.

In contrast, H_2_O_2_ treatment significantly increased MDA levels (Figure 6D), indicating enhanced lipid peroxidation (# *p* < 0.05). Pretreatment with PITT significantly reduced MDA accumulation in a concentration-dependent manner (* *p* < 0.05), suggesting that PITT alleviated membrane lipid peroxidation under oxidative stress.

Combined with the ROS results (Figure 5), these findings indicate that PITT protects RAW 264.7 cells against H_2_O_2_-induced oxidative injury by reducing intracellular oxidative burden and enhancing antioxidant defenses. This protective effect may be attributable to direct ROS scavenging and/or modulation of cellular antioxidant response pathways (e.g., Nrf2-related signaling), although further mechanistic studies are required to confirm the underlying mechanisms.

## 3. Materials and Methods

### 3.1. Chemicals and Materials

*Phellinus igniarius* samples used in this study were collected from Tonghua, Jilin Province, China. The RAW 264.7 cell line was obtained from the Cell Bank/Stem Cell Bank of the Chinese Academy of Sciences (Shanghai, China). Dulbecco’s Modified Eagle Medium (DMEM; VICMED, Xuzhou, Jiangsu, China) supplemented with 10% fetal bovine serum (FBS; VICMED, China) and 100 U/mL penicillin-streptomycin was used for cell culture. CCK-8, MDA, reduced glutathione (GSH), SOD, GSH-Px, and CAT assay kits were purchased from Shanghai Beyotime Biotechnology Co., Ltd. (Shanghai, China).

### 3.2. P. igniarius Terpenoid-Enriched Extract (PITT)

#### 3.2.1. Sample Preparation

The fruiting bodies of *P. igniarius* were dried at 45 °C for 48 h in a hot-air oven (DHG-9075A, Shanghai HuiTai, Shanghai, China) until constant weight. The dried samples were then ground into a fine powder using a high-speed grinder (JXFSTPRP-48, Shanghai JingXin, Shanghai, China). The powder was sieved through a 40-mesh sieve, weighed (ME204, Mettler Toledo, Zurich, Switzerland), and stored in a sealed container prior to further analysis.

#### 3.2.2. Determination of the Terpenoid Content

Total terpenoid content in *P. igniarius* was quantified according to the method of Zhang et al. [59] and used for subsequent optimization. Briefly, the sample solution (0.2 mL) was dried in a 70 °C water bath. Then, 0.2 mL of 5% (*w*/*v*) vanillin (purity ≥ 99%, Aladdin, Shanghai, China) in glacial acetic acid (purity ≥ 99%, Aladdin, Shanghai, China) and 1.8 mL of perchloric acid (purity ≥ 70%, ChengDu KeLong, Chengdu, China) were added. The mixture was incubated at 70 °C for 30 min and then rapidly cooled to room temperature in an ice-water bath. Glacial acetic acid was added to bring the total volume to 10 mL. Absorbance was measured at 552 nm using a UV-Vis spectrophotometer (UV1900 PC, ShangHai HaoTi, Shanghai, China). Total terpenoid yield was calculated using the following equation and expressed as mg ursolic acid equivalents per g dry weight (mg UAE/g DW).

$$\mathrm{Total}\;\mathrm{terpenoids}\;\mathrm{yield}=W(\mathrm{mg}/g)=\frac{C\times V\times D}{1000\times M}$$

where C is the terpenoid concentration determined from the ursolic acid standard curve (μg/mL), D is the dilution factor, V is the extract volume (mL), and M is the sample weight (g). Ursolic acid was used to generate the standard curve (Figure S1, Supporting Information). The regression equation was y = 0.0367x + 0.0073 (R^2^ = 0.9986), indicating good linearity.

It is worth noting that the vanillin–perchloric acid method is known to cross-react with sterols and fatty acids. Therefore, the measured ‘total terpenoids’ likely overestimates true terpenoid content. Moreover, the strong antioxidant activity cannot be attributed solely to terpenoids; phenolic co-extractives likely contribute substantially. This matrix effect is explicitly acknowledged.

#### 3.2.3. Single-Factor Test for PITT Extraction

*Phellinus igniarius* powder (1.0 g) was subjected to ultrasound-assisted (300 W, KQ-500DE, Kunshan Ultrasonic, Kunshan, China) extraction in an ethanol-water solution. The following factors were varied: ethanol concentration (65%, 70%, 75%, 80%, and 85%), liquid-to-solid ratio (1:10, 1:15, 1:20, 1:25, and 1:30, g/mL), extraction temperature (40, 50, 60, 70, and 80 °C), and extraction time (10, 15, 20, 25, and 30 min). The resulting extracts were filtered through a Büchner funnel. Each experiment was performed in triplicate, and data are presented as the mean ± standard deviation (SD).

#### 3.2.4. RSM Experimental Design

The extraction conditions for total terpenoids were optimized using a Box–Behnken design (BBD) at a fixed liquid-to-solid ratio of 20 mL/g. Extraction time (A: 15–25 min), extraction temperature (B: 50–70 °C), and ethanol concentration (C: 70–80%) were selected as independent variables, and total terpenoid content was used as the response. A response surface methodology (RSM) model was established to evaluate the effects of these variables and determine the optimal extraction conditions. The Box–Behnken design was selected because it requires fewer experimental runs than a central composite design while efficiently estimating quadratic models. Five center points were chosen to provide an independent estimate of pure error and to assess model lack-of-fit, which is critical for validating response surface curvature. Table S1 lists the experimental factors and their coded levels. Each design point was performed in triplicate, and results are presented as the mean. The optimal conditions were obtained using Design-Expert software (version 8.0; Stat-Ease Inc., Minneapolis, MN, USA). A value of *p* < 0.05 was considered statistically significant.

#### 3.2.5. LC-MS/MS Analysis

##### LC Conditions

PITT sample (100 mg) was extracted with 70% methanol (1000 μL), vortexed, and centrifuged at 12,000× *g* for 10 min at 4 °C. The supernatant was collected and stored for subsequent analysis. Chromatographic separation was performed using a Vanquish Flex UPLC system (Thermo Fisher Scientific, Bremen, Germany) equipped with a Zorbax Eclipse C18 column (1.8 μm, 2.1 mm × 100 mm; Agilent Technologies, Santa Clara, CA, USA). The column temperature was maintained at 30 °C, and the flow rate was set to 0.3 mL/min. The mobile phases were (A) water containing 0.1% formic acid and (B) acetonitrile. The gradient elution program is shown in Table S2.

##### Mass Spectroscopy (MS) Conditions

Electrospray ionization (ESI) was operated in both positive and negative ion modes. The sheath/auxiliary gas flow rates, ion transfer tube temperature, spray voltage, and heater temperature were set to 45/15 (arbitrary units), 330 °C, 3.5 kV, and 325 °C, respectively, for both modes. MS1 spectra were acquired in full-scan mode at a resolution of 120,000 over an m/z range of 100–1500. Data-dependent MS/MS (dd-MS2) spectra were acquired at a resolution of 60,000. High-energy collision dissociation (HCD) was used for fragmentation, with normalized collision energies (NCE) of 20, 40, and 60.

##### Qualitative Analysis of Metabolites

Compound Discoverer 3.3 was used for retention time alignment, peak detection, and peak extraction. Compounds were annotated based on MS/MS data by searching the Thermo mzCloud and Thermo mzVault databases. Relative abundances in the total triterpene extract were estimated based on peak areas.

### 3.3. Antioxidant Capacity In Vitro

#### 3.3.1. DPPH Radical Scavenging Capacity Assay

DPPH radical-scavenging activity was determined according to the method of Boateng and Yang, with slight modifications [60]. Briefly, 2 mL of sample solution at different concentrations was mixed with 2 mL of 0.2 mmol/L DPPH solution. The mixture was incubated for 30 min at room temperature in the dark, and the absorbance was measured at 517 nm. For the sample control, 2 mL of each sample solution was mixed with 2 mL of 95% ethanol. For the blank, 2 mL of 95% ethanol was mixed with 2 mL of 0.2 mmol/L DPPH solution. Vitamin C (Vc, purity ≥ 99%, Sigma-Aldrich, St. Louis, MO, USA) was used as the positive control. The DPPH radical-scavenging rate was calculated as follows:

$$\mathrm{DPPH}\;\mathrm{radical}\;\mathrm{scavenging}\;\mathrm{rate}\;(\%)\;=(1-\frac{A1-A2}{A0})\times 100$$

where A1 is the absorbance of the reaction mixture containing DPPH and the sample, A2 is the absorbance of the sample mixed with ethanol (sample control), and A0 is the absorbance of the DPPH solution mixed with ethanol (blank).

#### 3.3.2. ABTS•+ Radical Scavenging Capacity Assay

The assay was performed according to Arts et al. [61], with minor modifications. ABTS solution (7 mmol/L) was mixed with potassium persulfate solution (2.45 mmol/L) at a 1:1 (*v*/*v*) ratio and incubated for 16 h at room temperature in the dark. The resulting ABTS•+ working solution was diluted with phosphate buffer (pH 7.0) to an absorbance of 0.70 ± 0.02 at 734 nm. Sample solutions at different concentrations were mixed with the ABTS•+ working solution at a 1:20 (*v*/*v*) ratio. After incubation for 6 min at room temperature, the absorbance (A1) was measured at 734 nm. For the sample blank (A2), the ABTS•+ working solution was replaced with 95% ethanol under the same conditions. For the reagent blank (A0), the sample solution was replaced with 95% ethanol. The ABTS•+ radical scavenging rate was calculated as follows:

$$\mathrm{ABTS}•+\;\mathrm{radical}\;\mathrm{scavenging}\;\mathrm{rate}\;(\%)\;=\;(1-\frac{A1-A2}{A0})\times 100$$

#### 3.3.3. Ferric Reducing Antioxidant Power (FRAP)

Ferric reducing antioxidant power (FRAP) was determined according to Yang et al. [55], with minor modifications. The FRAP reagent was freshly prepared by mixing acetate buffer (300 mM, pH 3.6), FeCl_3_ (20 mM), and TPTZ (10 mM in 40 mM HCl) at a 10:1:1 (*v*/*v*/*v*) ratio. The FRAP reagent (180 µL) was mixed with the sample solution (20 µL) and incubated for 30 min at 37 °C. Absorbance was measured at 593 nm.

#### 3.3.4. Measurement of Hydroxyl Radical Scavenging Capacity

Hydroxyl radical scavenging activity was determined according to Ma et al. [62], with slight modifications. Briefly, 1 mL of sample solution at different concentrations was added to a test tube, followed by the addition of FeSO_4_ (6 mM, 1 mL), H_2_O_2_ (3%, 1 mL), and salicylic acid in ethanol (9 mM, 1 mL). The mixture was mixed thoroughly and incubated for 10 min at room temperature. It was then incubated in a 37 °C water bath for 30 min and cooled to room temperature. Absorbance was measured at 510 nm and recorded as A1. All measurements were performed in triplicate. For the control (A0), the sample solution was replaced with deionized water. For the background control (A2), the H_2_O_2_ solution was replaced with deionized water under the same conditions. Vitamin C (Vc) were used as positive controls. The hydroxyl radical scavenging rate was calculated as follows:

$$\mathrm{Hydroxyl}\;\mathrm{radical}\;\mathrm{scavenging}\;\mathrm{rate}\;(\%)\;=\;(1-\frac{A1-A2}{A0})\times 100$$

### 3.4. Antioxidant Activity in the Cell Model

It is hypothesized that the antioxidant activity of PITT may be related to structural features of the identified terpenoid subclasses. Sesquiterpenes (e.g., curdione, atractylenolide II) contain α,β-unsaturated lactone moieties, which could potentially act as Michael acceptors and may theoretically contribute to Nrf2 activation via Keap1 alkylation, although this was not directly tested. Triterpenes (e.g., betulin, soyasapogenol A) are more lipophilic and may primarily localize to membranes, where they could interrupt lipid peroxidation chain reactions. The potential additive or synergistic effects of these subclasses remain to be determined, and further studies are needed to validate these hypotheses.

#### 3.4.1. RAW 264.7 Macrophage Culture and Cell Viability Measurement of PITT

Cytotoxicity was evaluated according to Xu et al. [3], with slight modifications. RAW 264.7 cells were cultured in high-glucose DMEM supplemented with 10% heat-inactivated fetal bovine serum (FBS) and 1% penicillin-streptomycin (Solarbio Biotechnology Co., Ltd., Beijing, China). Cells were seeded into 96-well plates at a density of 1 × 10^4^ cells/mL (100 μL per well) and incubated overnight at 37 °C with 5% CO_2_. The medium was then replaced with 100 μL of DMEM containing PITT at different concentrations (0.01, 0.05, 0.1, 0.5, and 1.0 mg/mL). Cells treated with the same volume of DMEM served as the control. After incubation for 24 h, CCK-8 reagent (10 μL) was added to each well and incubated for 2 h. Absorbance at 450 nm was measured using a Synergy™ H1 microplate reader (BioTek, Winooski, VT, USA). Cell viability was calculated as follows:

Cell viability(%) = [(Asample − Ablank)/(Acontrol − Ablank)] × 100

#### 3.4.2. Cell Oxidative Damage Modeling

An oxidative injury model was established according to Liu et al. [57], with minor modifications. RAW 264.7 cells in the logarithmic growth phase were seeded into 96-well plates and incubated overnight to allow cell adhesion. The culture medium was then removed, and the cells were treated with DMEM containing different concentrations of H_2_O_2_ (0, 0.1, 0.2, 0.4, 0.6, 0.8, and 1.0 mmol/L) for 2, 4, or 6 h. Cell viability was subsequently assessed using the CCK-8 assay to determine the optimal H_2_O_2_ concentration and exposure time. The condition that reduced cell viability to 50–70% was selected as the standard injury condition for subsequent experiments. Excessively high viability may not produce measurable oxidative damage, whereas excessively low viability may cause irreversible injury, both of which are unfavorable for establishing a reliable oxidative stress model [63].

#### 3.4.3. Protection of PITT Against H_2_O_2_-Mediated Oxidative Injury

The protective effect of PITT against H_2_O_2_-induced oxidative injury was evaluated as previously described [55]. Briefly, RAW 264.7 cells were seeded into 24-well plates (500 µL per well) at a density of 2 × 10^4^ cells/mL and incubated for 24 h. The medium was then replaced with fresh medium containing PITT (10–1000 µg/mL) and incubated for 6 h. Subsequently, the medium was replaced with fresh medium containing H_2_O_2_ (0.6 mmol/L) and incubated for an additional 6 h. Cell viability was measured using the CCK-8 assay.

#### 3.4.4. ROS Assay in H_2_O_2_-Stimulate RAW 264.7 Cells

RAW 264.7 cells were seeded into 12-well plates containing coverslips (thickness: 0.13–0.16 mm; diameter: 20 mm) at a density of 5 × 10^4^ cells/well and incubated overnight. Cells were then incubated with PITT (0.01, 0.05, 0.1, and 0.2 mg/mL) for 4 h, followed by incubation with or without H_2_O_2_ (0.6 mmol/L) for 6 h. After incubation, a staining solution containing the fluorescent probe was added to each well according to the instructions of the Reactive Oxygen Species Assay Kit (Beyotime, Shanghai, China). To correct for potential autofluorescence of terpenoids, background fluorescence of unstained cells treated with PITT alone was measured and subtracted from each sample. Additionally, DCFH-DA fluorescence was confirmed to be independent of PITT autofluorescence by measuring signal in the absence of H_2_O_2_.

#### 3.4.5. Cellular Antioxidant Capacity Assay

After treating the cells as described in Section 2.4.3, the cells were collected and lysed. CAT, SOD, GSH-Px, MDA, and GSH levels in the cell lysates were measured according to the manufacturers’ instructions (Beyotime, Shanghai, China).

### 3.5. Statistical Analysis

All experiments were performed in triplicate. Data are presented as the mean ± standard deviation (SD). For the in vitro antioxidant assays, differences among groups were analyzed by one-way ANOVA followed by Tukey’s post hoc test using GraphPad Prism 8. A value of *p* < 0.05 was considered statistically significant.

## 4. Conclusions

In this study, response surface methodology (RSM) was used to optimize the extraction of total terpenoids from *Phellinus igniarius* (PITT). The optimal conditions were an extraction temperature of 64 °C, an extraction time of 21 min, and an ethanol concentration of 78%, yielding a validated total terpenoid content of 7.078 mg/g. The optimized extraction conditions were selected not only for maximizing yield but also for preserving terpenoid integrity. Moderate temperature (64 °C) avoids thermal degradation observed above 70 °C, while the short extraction time (21 min) minimizes oxidative exposure. The 78% ethanol–water mixture provides a stabilizing polar environment for oxygenated terpenoids and limits co-extraction of pro-oxidant lipids. These conditions thus balance extraction efficiency with compound stability, supporting the practical application of the RSM model for scale-up and storage of PITT. LC-MS/MS profiling further revealed several putative bioactive constituents in PITT, including curdione and betulin. The present study used *P. igniarius* fruiting bodies collected from a single geographic location (Tonghua, Jilin Province, China). Feedstock variability—including differences in harvest season, tree host species (e.g., *Populus* vs. *Betula*), fruiting body age, and post-harvest storage conditions—may influence both terpenoid content and extraction efficiency. For example, terpenoid biosynthesis in medicinal fungi can be affected by environmental stress and substrate composition. Therefore, the optimal conditions reported here should be considered specific to the tested batch. Scale-up or application to *P. igniarius* from different origins may require re-optimization. Future studies should assess batch-to-batch variability and consider metabolomic fingerprinting to standardize raw material quality. Bioactivity assays demonstrated that PITT exhibited concentration-dependent antioxidant activity in vitro and alleviated H_2_O_2_-induced oxidative stress in RAW 264.7 cells. Overall, these findings provide a feasible technical basis for scaling up terpenoid extraction from *P. igniarius* and support the potential application of PITT as a natural antioxidant ingredient in functional foods and related products. However, it should be noted that total terpenoids accounted for less than 1% and LC-MS/MS profiling revealed signals corresponding to non-terpenoid compounds (e.g., phenolics), which are known co-extractives in *Phellinus igniarius*. Comprehensive identification of these non-terpenoid components was not performed in the present study. Future studies should characterize the non-terpenoid fraction to dissect its individual contribution to the observed antioxidant activity and to evaluate potential synergistic effects between terpenoids and other bioactive constituents.

## Figures and Tables

**Table 1:** ANOVA outcomes for quadratic regression models.

Factor	Sum of Squares	DF	Mean Square	*F*-Value	*p*-Value
Model	7.62	9	0.85	213.65	<0.0001
A	3.511 × 10^−4^	1	3.511 × 10^−4^	0.089	0.7746
B	0.92	1	0.92	232.84	<0.0001
C	0.80	1	0.80	202.53	<0.0001
AB	1.000 × 10^−4^	1	1.000 × 10^−4^	0.025	0.8783
AC	4.225 × 10^−5^	1	4.225 × 10^−5^	0.011	0.9207
BC	1.122 × 10^−3^	1	1.122 × 10^−3^	0.28	0.6111
A^2^	0.98	1	0.98	246.73	<0.0001
B^2^	1.03	1	1.03	259.17	<0.0001
C^2^	3.34	1	3.34	841.86	<0.0001
Residual	0.028	7	3.963 × 10^−3^		
Lack of fit	9.714 × 10^−3^	3	3.238 × 10^−3^	0.72	0.5908
Pure error	0.018	4	4.507 × 10^−3^		
Cor. total	7.65	16			

**Table 2:** Major terpenoids identified in PITT by LC-MS/MS.

Name	RT (min)	Molecular Formula	Theoretical [M+H]^+^	Observed [M+H]^+^	Mass Error (ppm)	MS/MS Fragment (+)	MS/MS Fragment (−)	References
Curdione	10.747	C_15_ H_24_ O_2_	236.17763	236.17757	−0.25	MS^2^[236]: 219.17, 191.11, 177.13, 163.11, 95.08, 81.06.		[23,24,25,26]
linalyl isovalerate	14.075	C_15_ H_26_ O_2_	238.19328	238.19253	−3.16		MS^2^[238]:154.58, 88.37, 82.29, 70.24, 59.42	
Atractylenolide II	13.121	C_15_ H_20_ O_2_	232.14633	232.1463	−0.12	MS^2^[232]: 205.16, 187.15, 171.12, 159.12, 145.10		[27,28,29,30,31]
2-(8-Hydroxy-4a,8-dimethyldecahydro-2-naphthalenyl)acrylic acid	10.4	C_15_ H_24_ O_3_	252.17254	252.17199	−2.22	MS^2^[252]: 235.17, 207.17, 177.13, 95.09, 81.07		
4-Hydroxy-4a,8-dimethyl-3-methylene-3,3a,4,4a,7a,8,9,9a-octahydroazuleno[6,5-b]furan-2,5-dione	10.359	C_15_ H_18_ O_4_	262.12051	262.12007	−1.68		MS^2^[262]: 217.12, 171.11, 149.06, 119.05, 112.01, 80.14, 65.4	
3,7,15-Trihydroxy-12,13-epoxytrichothec-9-en-8-one	6.846	C_15_ H_20_ O_6_	296.12599	296.1254	−2		MS^2^[296]: 249.11 (100%), 277.10, 187.11, 163.08, 133.06, 107.05, 73.03, 57.03	
Culmorin	14.077	C_15_ H_26_ O_2_	238.19328	238.19327	−0.06	MS^2^[238]: 222.19, 203.18, 183.14, 179.18, 147.12, 123.12		
6-Hydroxy-5a,9-dimethyl-3-methylene-3a,4,5,5a,6,7,9a,9b-octahydronaphtho[1,2-b]furan-2(3H)-one	10.292	C_15_ H_20_ O_3_	248.14124	248.14124	0	MS^2^[248]: 231.14, 213.13, 203.14, 185.13, 175.08, 147.08, 137.06, 123.12, 95.09, 93.07		
Costunolide	11.16	C_15_ H_20_ O_2_	232.14633	232.14631	−0.09	MS^2^[232]: 187.15, 173.13, 159.12, 145.10, 131.09		[32,33,34]
Verrucarol	10.294	C_15_ H_22_ O_4_	266.15181	266.15167	−0.52	MS^2^[266]: 249.11, 231.10, 203.11, 185.10, 161.06		
Abscisic acid	10.642	C_15_ H_20_ O_4_	264.13616	264.13563	−2		MS^2^[264]: 245.12, 219.14, 287.61, 185.33, 165.25, 129.26, 108.76, 111.02	[35,36]
Isoalantolactone	9.86	C_15_ H_20_ O_2_	232.14633	232.14629	−0.17	MS^2^[232]: 215.14, 187.15, 173.13, 159.12, 145.10, 131.09, 95.09		[37,38]
Ageratriol	11.015	C_15_ H_24_ O_3_	252.17254	252.17244	−0.41	MS^2^[252]: 217.16, 189.16, 175.15, 153.13, 135.12, 133.1, 123.12, 109.1		
Camphor	13.471	C_10_ H_16_ O	152.12012	152.12017	0.36	MS^2^[152]: 135.12, 107.09, 95.09, 93.07, 81.07, 69.07		[39,40]
Labdanolic acid	16.526	C_20_ H_36_ O_3_	324.26644	324.26603	−1.28		MS^2^[324]: 302.89, 277.22, 240.36, 179.14, 141.24, 126.79, 111.12	[41]
Azuleno(5,6-c)furan-1(3H)-one,4,4a,5,6,7,7a,8,9-octahydro-3,4,8-trihydroxy-6,6,8-trimethyl-	6.929	C_15_ H_22_ O_5_	282.14672	282.14623	−1.75		MS^2^[282]: 263.15, 237.13, 219.14, 205.12, 177.11, 133.03, 99.04	
Nootkatone	13.029	C_15_ H_22_ O	218.16707	218.16708	0.06	MS^2^[218]: 203.14, 201.13, 177.11, 161.10, 135.12, 125.06		[42,43,44]
Betulin	15.18	C_30_ H_50_ O_2_	442.38108	442.38083	−0.58	MS^2^[442]: 425.38, 407.37, 357.31, 298.23, 285.18, 203.18, 163.15, 109.1		[45,46]
Soyasapogenol A	17.026	C_30_ H_50_ O_4_	474.37091	474.37083	−0.16	MS^2^[474]: 457.37, 439.36, 411.36, 229.19, 149.13, 121.1, 109.1, 95.06		[47]
Lupenone	17.45	C_30_ H_48_ O	424.37052	424.37023	−0.68	MS^2^[424]: 407.37, 390.28, 245.15, 187.11, 163.15, 135.12, 107.09		[48,49]

Note: All compounds were tentatively identified based on LC-MS/MS data (mass error < 5 ppm, MS/MS fragmentation) using Compound Discoverer 3.3 (Thermo mzCloud and mzVault). Authentic standards were not available for confirmation. RT indicates retention time.

## Data Availability

Data will be made available on request.

## Supplementary Materials

The following supporting information can be downloaded at: https://www.mdpi.com/article/10.3390/molecules31111929/s1, Figure S1: Ursolic acid standard curve. Figure S2: Total ion chromatograms. A: negative- and B: positive-ion modes.; Table S1: Factors and levels of BBD for the extraction of PITT. Table S2: Liquid chromatography mobile phase conditions. Table S3: Design and findings of the response surface test. Table S4: Representative MS/MS spectra for each compound.

## References

[1] Lushchak V.I. (2014). Free radicals, reactive oxygen species, oxidative stress and its classification. Chem. Biol. Interact..

[2] Damiano S., Longobardi C., Iervolino V., Florio S., Giordano A., Pelagalli A., Avagliano C., Lauritano C., Ciarcia R. (2025). Antioxidant and antiproliferative potential activity of the marine carotenoid fucoxanthin in the treatment of chronic blood cancer. J. Funct. Foods.

[3] Xu P., Bai B., Chen H., Zhang Y., Ma Z., Wu G., Zhou Y., Zhu K. (2026). A polysaccharide from *Artocarpus heterophyllus* Lam. pulp ameliorates oxidative stress and lipid accumulation in HepG2 cells. J. Funct. Foods.

[4] Zapora E., Wolkowycki M., Bakier S., Zjawiony J.K. (2016). *Phellinus igniarius*: A pharmacologically active polypore mushroom. Nat. Prod. Commun..

[5] Cai C., Ma J., Han C., Jin Y., Zhao G., He X. (2019). Extraction and antioxidant activity of total triterpenoids in the mycelium of a medicinal fungus, *Sanghuangporus sanghuang*. Sci. Rep..

[6] Gu X., Hao D., Xiao P. (2022). Research progress of Chinese herbal medicine compounds and their bioactivities: Fruitful 2020. Chin. Herb. Med..

[7] Ni Z., Li J., Qian X., Yong Y., Wu M., Wang Y., Lv W., Zhang S., Zhang Y., Shao Y. (2023). *Phellinus igniarius* Polysaccharides Ameliorate Hyperglycemia by Modulating the Composition of the Gut Microbiota and Their Metabolites in Diabetic Mice. Molecules.

[8] Li H., Zhang X., Gu L., Li Q., Ju Y., Zhou X., Hu M., Li Q. (2022). Anti-Gout Effects of the Medicinal Fungus *Phellinus igniarius* in Hyperuricaemia and Acute Gouty Arthritis Rat Models. Front. Pharmacol..

[9] Lung M.Y., Tsai J.C., Huang P.C. (2010). Antioxidant Properties of Edible Basidiomycete *Phellinus igniarius* in Submerged Cultures. J. Food Sci..

[10] Xie A., He F., Wang L., Shi W., Xu P., Wang T., Li P. (2025). *Phellinus igniarius* alcohol extract restores the immune homeostasis in cyclophosphamide induced immunocompromised mice. J. Funct. Foods.

[11] Zhu X., Guo R., Su X., Shang K., Tan C., Ma J., Zhang Y., Lin D., Ma Y., Zhou M. (2023). Immune-enhancing activity of polysaccharides and flavonoids derived from *Phellinus igniarius* YASH1. Front. Pharmacol..

[12] Wang C.-Y., Chen Y.-W., Hou C.-Y. (2019). Antioxidant and antibacterial activity of seven predominant terpenoids. Int. J. Food Prop..

[13] Wang Y., Zhang S., Ma Y., Du X., Zong Q., Lin D., Lai M., Huang T., Luo Q., Yang L. (2024). Solvent effects on terpenoid compositions and antioxidant activities of *Cinnamomum camphora* (L.) J. Presl extracts and the main antioxidant agent evaluation through in vitro and in vivo assay. Chem. Biol. Technol. Agric..

[14] Zhou X., Shi Q., Li J., Quan S., Zhang X., Gu L., Li H., Ju Y., Hu M., Li Q. (2022). Medicinal fungus *Phellinus igniarius* alleviates gout in vitro by modulating TLR4/NF-kB/NLRP3 signaling. Front. Pharmacol..

[15] Dong Y., Wang T., Gan B., Wasser S.P., Zhang Z., Zhao J., Duan X., Cao L., Feng R., Miao R. (2024). Antioxidant activity of *Phellinus igniarius* fermentation mycelia contributions of different solvent extractions and their inhibitory effect on α-amylase. Heliyon.

[16] Zhang H., Ma H., Liu W., Pei J., Wang Z., Zhou H., Yan J. (2014). Ultrasound enhanced production and antioxidant activity of polysaccharides from mycelial fermentation of *Phellinus igniarius*. Carbohydr. Polym..

[17] Azwanida N.N. (2015). A Review on the Extraction Methods Use in Medicinal Plants, Principle, Strength and Limitation. Med. Aromat. Plants.

[18] Zheng S., Zhang W., Liu S. (2020). Optimization of ultrasonic-assisted extraction of polysaccharides and triterpenoids from the medicinal mushroom *Ganoderma lucidum* and evaluation of their in vitro antioxidant capacities. PLoS ONE.

[19] Wan C., Tong Z., Yang J., Tao R., Zhao Z., Sai M., Meng X., Xiao K., Wang Q., Ma H. (2025). Ultrasound-assisted extraction, enrichment, and hypolipidemic potential of triterpenes from *Grifola frondosa* mycelia. BMC Chem..

[20] Wu J., Zhang Z., Lan H., Sun T., Li Z., Hung W., Hu H., Yang Z., Zhang J. (2025). Response surface analysis and anti-inflammatory activity of a potential postbiotic exopolysaccharide produced by probiotic *Lacticaseibacillus paracasei* K56. J. Funct. Foods.

[21] Ye Z., Wang W., Yuan Q., Ye H., Sun Y., Zhang H., Zeng X. (2016). Box-Behnken design for extraction optimization, characterization and in vitro antioxidant activity of *Cicer arietinum* L. hull polysaccharides. Carbohydr. Polym..

[22] Wu S.H., Zhou L.W., Dai Y.C., Khojimatov O.K., Gafforov Y., Bussmann R.W. (2023). *Sanghuangporus lonicerinus* (Bondartsev). Ethnobiology of Uzbekistan (Ethnomedicinal Knowledge of Mountain Communities).

[23] Chen Y., Zhu Z., Chen J., Zheng Y., Limsila B., Lu M., Gao T., Yang Q., Fu C., Liao W. (2021). Terpenoids from Curcumae Rhizoma: Their anticancer effects and clinical uses on combination and versus drug therapies. Biomed. Pharmacother..

[24] Chi B.J., Duan Z.L., Hasan A., Yin X.Z., Cui B.Y., Wang F.F. (2024). Effect and Mechanism of Curdione Combined with Gemcitabine on Migration and Invasion of Bladder Cancer. Biochem. Genet..

[25] Ma Y., Wang P., Wu Z., Li M., Gu Y., Wu H., Liu H. (2023). Curdione Relieved Isoproterenol-Induced Myocardial Damage through Inhibiting Oxidative Stress and Apoptosis. Am. J. Chin. Med..

[26] Zhang P., Liu H., Yu Y., Peng S., Zhu S. (2024). Role of Curcuma longae Rhizoma in medical applications: Research challenges and opportunities. Front. Pharmacol..

[27] Tian S., Yu H. (2017). Atractylenolide II Inhibits Proliferation, Motility and Induces Apoptosis in Human Gastric Carcinoma Cell Lines HGC-27 and AGS. Molecules.

[28] Wang J., Nasser M.I., Adlat S., Ming Jiang M., Jiang N., Gao L. (2018). Atractylenolide II Induces Apoptosis of Prostate Cancer Cells through Regulation of AR and JAK2/STAT3 Signaling Pathways. Molecules.

[29] Xiao C., Xu C., He N., Liu Y., Wang Y., Zhang M., Ji K., Du L., Wang J., Wang Q. (2020). Atractylenolide II prevents radiation damage via MAPKp38/Nrf2 signaling pathway. Biochem. Pharmacol..

[30] Zhang Y., Liu Y., Wang J., Jiang Z., Zhang L., Cui Y., Zhao D., Wang Y. (2022). Atractylenolide II inhibits tumor-associated macrophages (TAMs)-induced lung cancer cell metastasis. Immunopharmacol. Immunotoxicol..

[31] Lin Y., Chen K., Zhu M., Song W., Wu G., Pan A. (2024). Atractylenolide II regulates the proliferation, ferroptosis, and immune escape of hepatocellular carcinoma cells by inactivating the TRAF6/NF-kappaB pathway. Naunyn Schmiedebergs Arch. Pharmacol..

[32] Kim D.Y., Choi B.Y. (2019). Costunolide—A Bioactive Sesquiterpene Lactone with Diverse Therapeutic Potential. Int. J. Mol. Sci..

[33] Li Q., Wang Z., Xie Y., Hu H. (2020). Antitumor activity and mechanism of costunolide and dehydrocostus lactone: Two natural sesquiterpene lactones from the Asteraceae family. Biomed. Pharmacother..

[34] Wang X., Zhang L., Huang K., Lou C., Xia Y., Zhou Y., Wen W. (2025). Costunolide Reduces DN Inflammatory Response and Renal Thrombosis by Inhibiting NET Formation. J. Diabetes Res..

[35] Zhao C.C., Xu J., Xie Q.M., Zhang H.Y., Fei G.H., Wu H.M. (2021). Abscisic acid suppresses the activation of NLRP3 inflammasome and oxidative stress in murine allergic airway inflammation. Phytother. Res..

[36] Chen X., Ding C., Liu W., Liu X., Zhao Y., Zheng Y., Dong L., Khatoon S., Hao M., Peng X. (2021). Abscisic acid ameliorates oxidative stress, inflammation, and apoptosis in thioacetamide-induced hepatic fibrosis by regulating the NF-κB signaling pathway in mice. Eur. J. Pharmacol..

[37] Hu F., Yang P. (2022). Isoalantolactone exerts anticancer effects on human HEC-1-B endometrial cancer cells via induction of ROS mediated apoptosis and inhibition of MEK/ERK signalling pathway. Acta Biochim. Pol..

[38] Zhou C., Chen J., Liu K., Maharajan K., Zhang Y., Hou L., Li J., Mi M., Xia Q. (2023). Isoalantolactone protects against ethanol-induced gastric ulcer via alleviating inflammation through regulation of PI3K-Akt signaling pathway and Th17 cell differentiation. Biomed. Pharmacother..

[39] Shabbir A., Parvinzadeh Gashti M. (2026). Camphor’s Therapeutic Uses and Potential Hazards: An In-Depth Review of Its Medicinal Applications. Molecules.

[40] Li Z., Gan Y., Kang T., Zhao Y., Huang T., Chen Y., Liu J., Ke B. (2023). Camphor Attenuates Hyperalgesia in Neuropathic Pain Models in Mice. J. Pain. Res..

[41] Martins I., Varela A., Frija L.M.T., Estevão M.A.S., Planchon S., Renaut J., Afonso C.A.M., Silva Pereira C. (2017). Proteomic Insights on the Metabolism of *Penicillium janczewskii* during the Biotransformation of the Plant Terpenoid Labdanolic Acid. Front. Bioeng. Biotechnol..

[42] Bezerra Rodrigues Dantas L., Silva A.L.M., da Silva Júnior C.P., Alcântara I.S., Correia de Oliveira M.R., Oliveira Brito Pereira Bezerra Martins A., Ribeiro-Filho J., Coutinho H.D.M., Rocha Santos Passos F., Quintans-Junior L.J. (2020). Nootkatone Inhibits Acute and Chronic Inflammatory Responses in Mice. Molecules.

[43] Al-Salam S., Kandhan K., Sudhadevi M., Tariq S. (2022). Nootkatone Ameliorates Doxorubicin Induced Myocardial Injury through Modulation of NF-κB Signals and Oxidative Stress. Cell. Physiol. Biochem..

[44] You Y.-L., Choi H.-S. (2024). Nootkatone (NK), a grapefruit-derived aromatic compound, inhibited lipid accumulation by regulating JAK2-STAT signaling and antioxidant response in adipocyte. Food Sci. Biotechnol..

[45] Li Z., Huang M., Wang J., Lin J., Wu Q., Wang P., Zhuo S., Zhang W., Liu Z., Deng Q. (2025). Betulin, an active component from Chinese herb birch bark, suppresses tumor angiogenesis and tumor growth by inhibiting the PAX2/VEGF-A/VEGR2 signaling pathway in non-small cell lung cancer. Phytomedicine.

[46] Lu X., Yang S., Lu Q., Zhang Y., Cha Z., Huang W., Li T. (2024). Betulin ameliorates neuronal apoptosis and oxidative injury via DJ-1/Akt/Nrf2 signaling pathway after subarachnoid hemorrhage. CNS Neurosci. Ther..

[47] Omar A., Kalra R.S., Putri J., Elwakeel A., Kaul S.C., Wadhwa R. (2020). Soyasapogenol-A targets CARF and results in suppression of tumor growth and metastasis in p53 compromised cancer cells. Sci. Rep..

[48] Li F., Sun X., Sun K., Kong F., Jiang X., Kong Q. (2024). Lupenone improves motor dysfunction in spinal cord injury mice through inhibiting the inflammasome activation and pyroptosis in microglia via the nuclear factor kappa B pathway. Neural Regen. Res..

[49] Xu F., Huang X., Wu H., Wang X. (2018). Beneficial health effects of lupenone triterpene: A review. Biomed. Pharmacother..

[50] Chen L.G., Jan Y.S., Tsai P.W., Norimoto H., Michihara S., Murayama C., Wang C.C. (2016). Anti-inflammatory and Antinociceptive Constituents of *Atractylodes japonica* Koidzumi. J. Agric. Food Chem..

[51] Bentharavithana J., Islam T., Xu B. (2025). Medicinal Mushrooms in Colon Cancer Therapy: Mechanisms of Action of Bioactive Compounds and Therapeutic Potential. Int. J. Mol. Sci..

[52] Zhang H., Yuan B., Huang H., Qu S., Yang S., Zeng Z. (2018). Gastrodin induced HO-1 and Nrf2 up-regulation to alleviate H_2_O_2_-induced oxidative stress in mouse liver sinusoidal endothelial cells through p38 MAPK phosphorylation. Braz. J. Med. Biol. Res..

[53] Wu Z., Wang H., Fang S., Xu C. (2018). Roles of endoplasmic reticulum stress and autophagy on H_2_O_2_-induced oxidative stress injury in HepG2 cells. Mol. Med. Rep..

[54] Chao W.-W., Chung Y.-C., Shih I.P., Wang H.-Y., Chou S.-T., Hsu C.-K. (2015). Red Bean Extract Inhibits Lipopolysaccharide-Induced Inflammation and H_2_O_2_-Induced Oxidative Stress in RAW 264.7 Macrophages. J. Med. Food.

[55] Yang Y., Xu S., Yang K., Sun Y., Yang R., Hu Y., Chen G., Cai H. (2023). Characterization and In Vitro Antioxidant and Anti-Inflammatory Activities of Ginsenosides Extracted from Forest-Grown Wild *Panax quinquefolius* L.. Foods.

[56] Vuong L.D., Nguyen Q.N., Truong V.L. (2019). Anti-inflammatory and anti-oxidant effects of combination between sulforaphane and acetaminophen in LPS-stimulated RAW 264.7 macrophage cells. Immunopharmacol. Immunotoxicol..

[57] Liu D., Quan R., Wu X., Chong Z., Zhang J., Liu R., Han J., Yang Y. (2025). Extraction, structural characterization and in vitro antioxidant activity of porcine small intestinal glycoproteins from heparin processing wastewater. J. Funct. Foods.

[58] Li H., He J., Yang X., Li X., Luo D., Wei C., Ma J., Zhang Y., Yang J., Zhang X. (2016). Glutathione-dependent induction of local and systemic defense against oxidative stress by exogenous melatonin in cucumber (*Cucumis sativus* L.). J. Pineal Res..

[59] Zhang W., Cai Y., Chen X., Ji T., Sun L. (2020). Optimized extraction based on the terpenoids of *Heterotrigona itama* propolis and their antioxidative and anti-inflammatory activities. J. Food Biochem..

[60] Boateng I.D., Yang X. (2024). Water-Soluble Intracellular Polysaccharides (IPSW-2 to 4) from *Phellinus igniarius* Mycelia: Fractionation, Structural Elucidation, and Antioxidant Activity. Foods.

[61] Arts M.J.T.J., Haenen G.R.M.M., Voss H.-P., Bast A. (2004). Antioxidant capacity of reaction products limits the applicability of the Trolox Equivalent Antioxidant Capacity (TEAC) assay. Food Chem. Toxicol..

[62] Ma T., Hu N., Ding C., Zhang Q., Li W., Suo Y., Wang H., Bai B., Ding C. (2016). In vitro and in vivo biological activities of anthocyanins from *Nitraria tangutorun* Bobr. fruits. Food Chem..

[63] Zhao X.-C., Zhang L., Yu H.-X., Sun Z., Lin X.-F., Tan C., Lu R.-R. (2011). Curcumin protects mouse neuroblastoma Neuro-2A cells against hydrogen-peroxide-induced oxidative stress. Food Chem..

