# Supplementary material for: Optimization of Extraction of Total Terpenoids from Phellinus igniarius Using Response Surface Methodology and Evaluation of Their Antioxidant Activity
Source: Molecules. 2026 Jun 3;31(11):1929. doi: 10.3390/molecules31111929 (PMC13257707; doi:10.3390/molecules31111929)
Supplement: Supplementary file 1 [file molecules-31-01929-s001.zip › molecules-4326373-supplementary.pdf]

Optimization of Extraction of Total Terpenoids from *Phellinus igniarius* Using Response Surface Methodology and Evaluation of Their Antioxidant Activity

Supplementary material

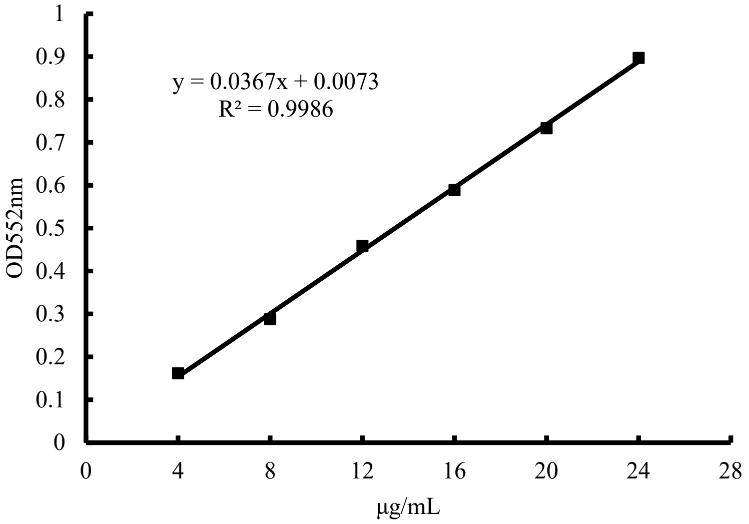

Figure S1. Ursolic acid standard curve.

Table S1. Factors and levels of BBD for the extraction of PITT.

Level	Factor		
	A: extraction time(min)	B: extraction temperature(°C)	C:alcohol concentration(%)
-1	15	50	70
0	20	60	75
1	25	70	80

Table S2. Liquid chromatography mobile phase conditions.

Time(min)	Flow rate(μL/min)	Gradient	B%Acetonitrile	A%Fomic acid
0-2	300	-	5	95
2-6	300	Linear gradient	30	70

6-7	300	-	30	70
7-12	300	Linear gradient	78	22
12-14	300	-	78	22
14-17	300	Linear gradient	95	5
17-20	300	-	95	5
20-21	300	Linear gradient	5	95
21-25	300	-	5	95

Table S3. Design and findings of the response surface test.

Run	A	B	C	Yield(mg/g)
1	20	60	75	7.024
2	15	70	75	6.392
3	20	70	70	5.657
4	20	60	75	6.893
5	15	60	80	5.982
6	20	60	75	7.003
7	20	60	75	7.065
8	20	50	70	5.021
9	20	50	80	5.553
10	15	50	75	5.693
11	25	70	75	6.357
12	25	60	80	5.987
13	15	60	70	5.287
14	20	60	75	7.044
15	25	50	75	5.678
16	25	60	70	5.279
17	20	70	80	6.256

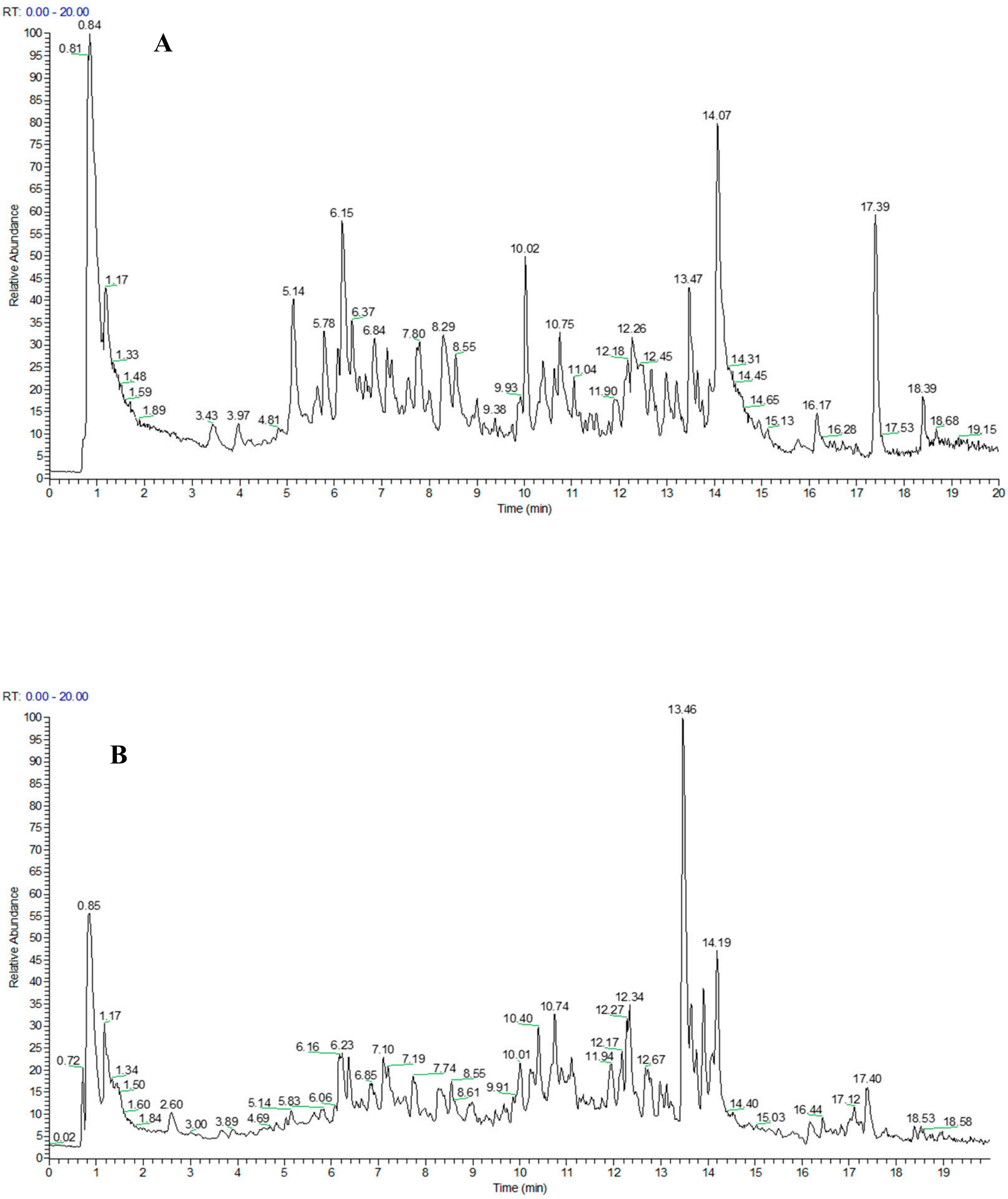

Figure S2. Total ion chromatograms. A: negative- and B: positive-ion modes.

Table S4. Representative MS/MS spectra for each compound.

<table>
<tr><th>Name</th><th>MS/MS spectra</th></tr>
<tr><td>Curdione</td><td>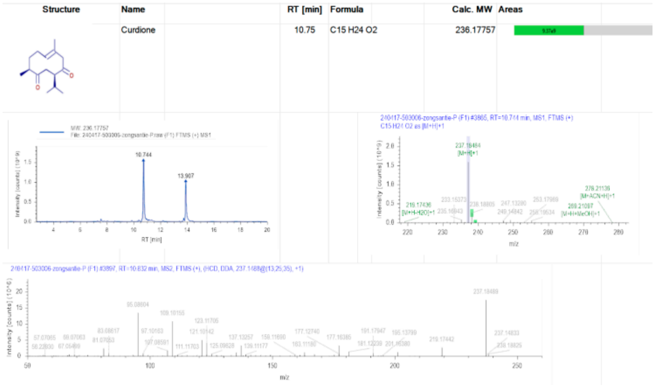
</td></tr>
<tr><td>linalyl isovalerate</td><td>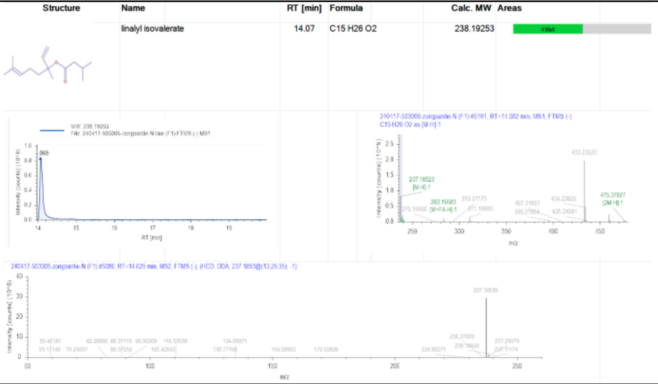
</td></tr>
<tr><td>Atractylenolide II</td><td>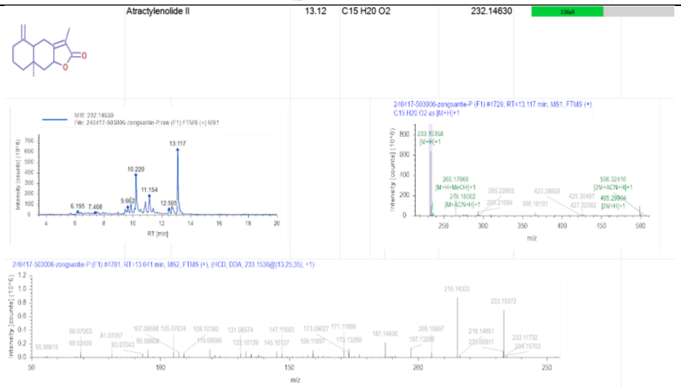
</td></tr>
<tr><td>2-(8-Hydroxy-4a,8-dimethyldecahydro-2-naphthalenyl)acrylic acid</td><td>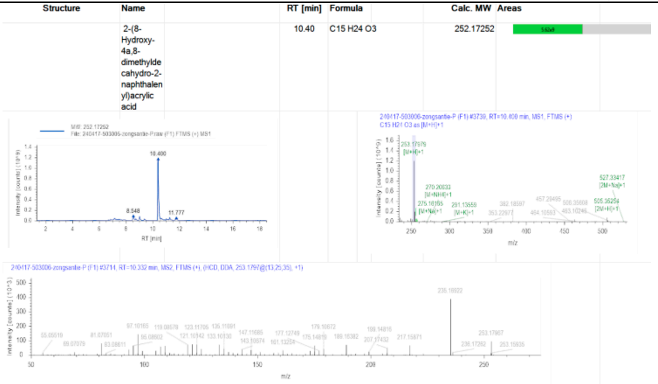
</td></tr>
</table>

4-Hydroxy-4a,8-dimethyl-3-methylene-3,3a,4,4a,7a,8,9,9a-octahydroazuleno[6,5-b]furan-2,5-dione	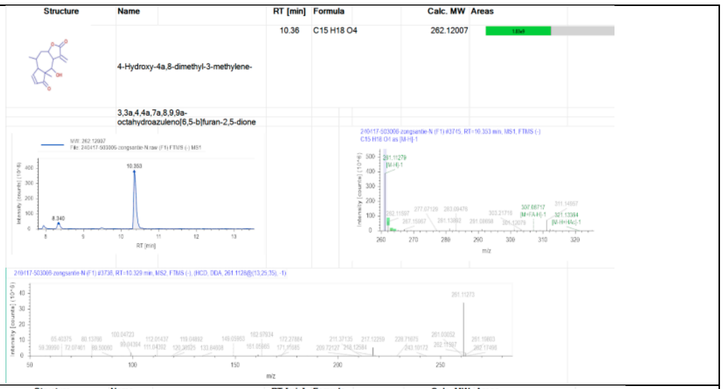
3,7,15-Trihydroxy-12,13-epoxytrichothec-9-en-8-one	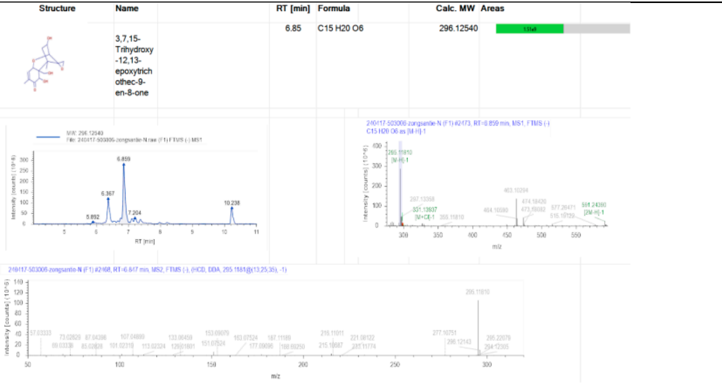
Culmorin	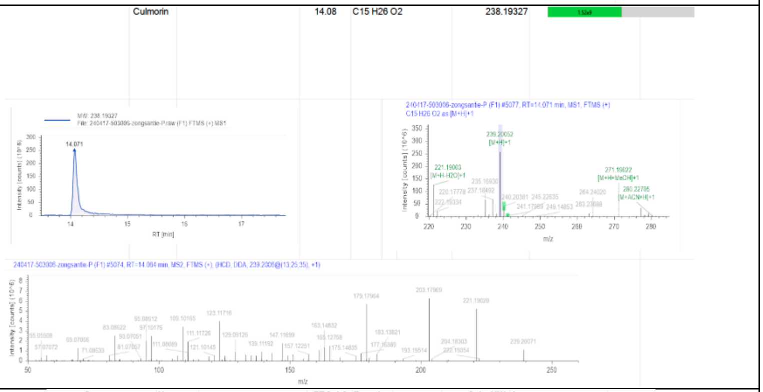
6-Hydroxy-5a,9-dimethyl-3-methylene-3a,4,5,5a,6,7,9a,9b-octahydronaphtho[1,2-b]furan-2(3H)-one	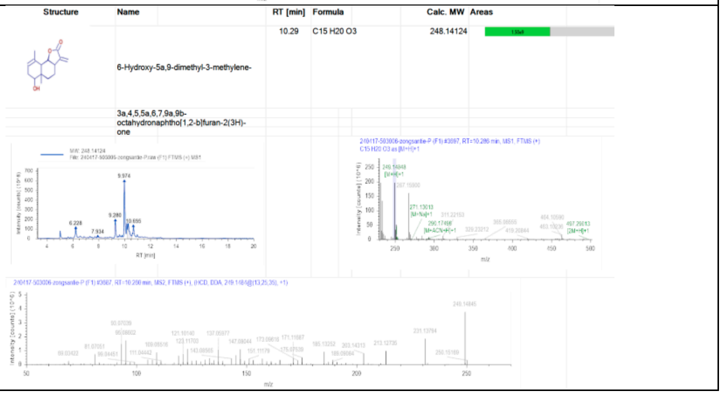

Costunolide	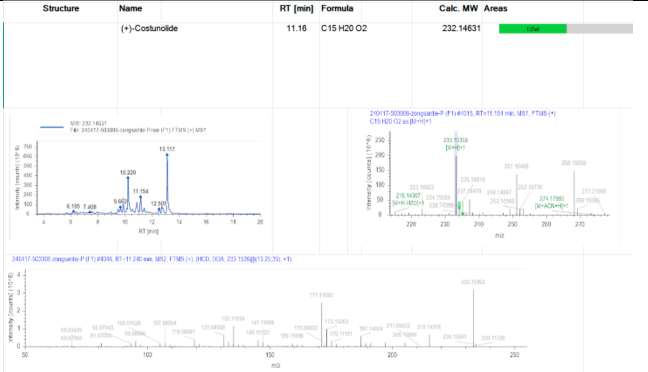
Verrucarol	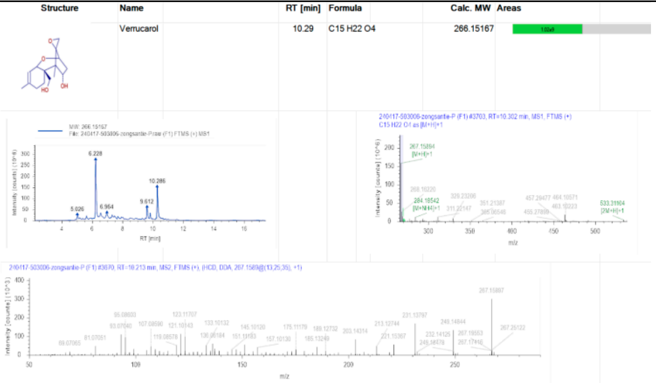
Abscisic acid	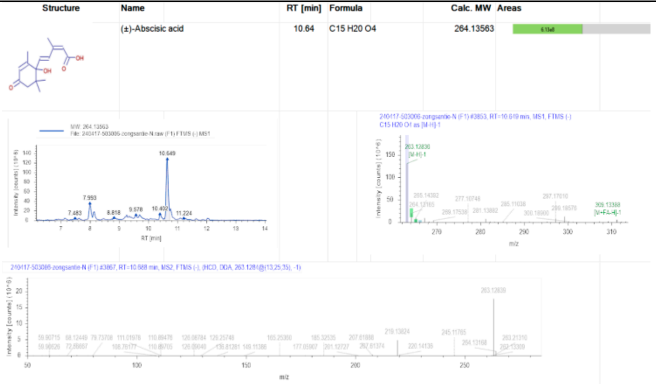
Isoalantolactone	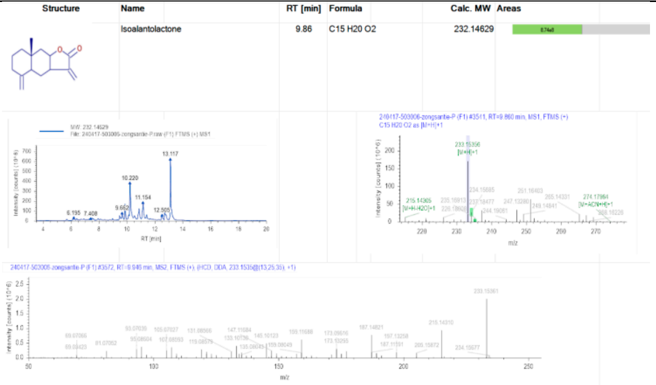

<table>
<tr><td>Ageratriol</td><td>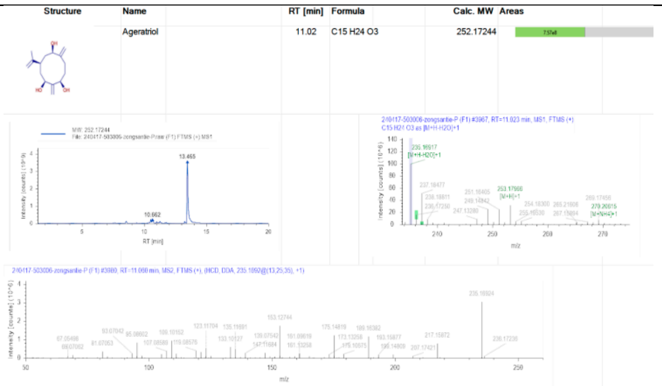
</td></tr>
<tr><td>Camphor</td><td>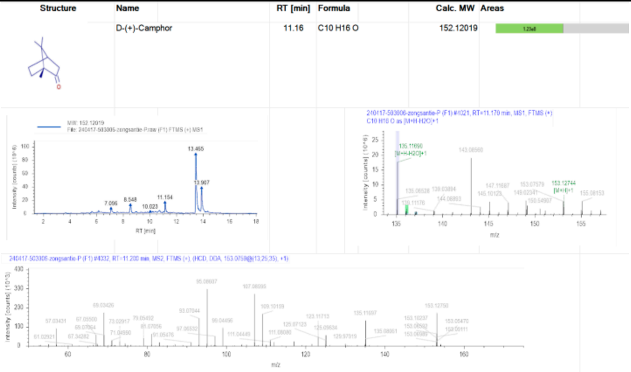
</td></tr>
<tr><td>Labdanolic acid</td><td>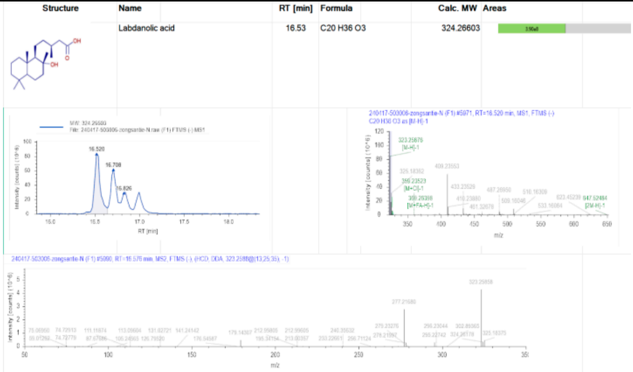
</td></tr>
<tr><td>Azuleno(5,6-c)furan-1(3H)-one,4,4a,5,6,7,7a,8,9-octahydro-3,4,8-trihydroxy-6,6,8-trimethyl-</td><td>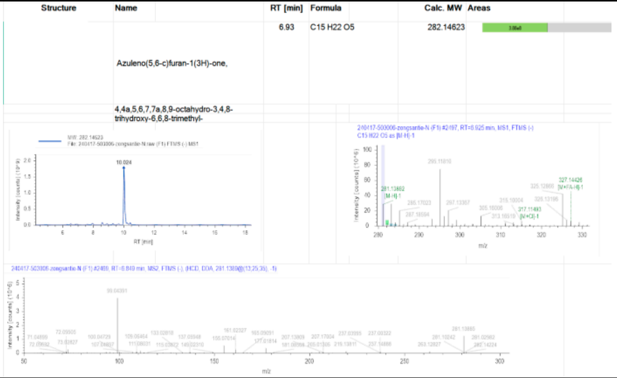
</td></tr>
</table>

<table>
<tr><td>Nootkatone</td><td>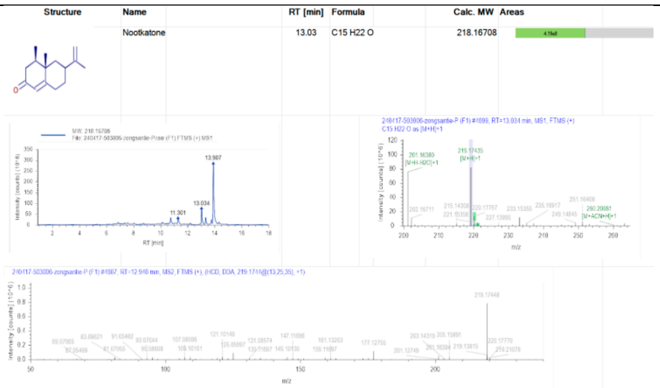
</td></tr>
<tr><td>Betulin</td><td>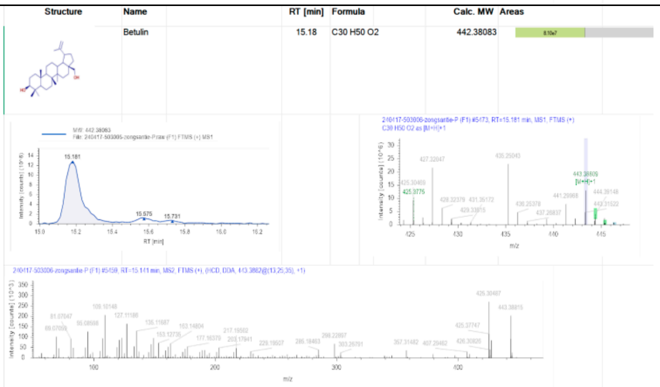
</td></tr>
<tr><td>Soyasapogenol A</td><td>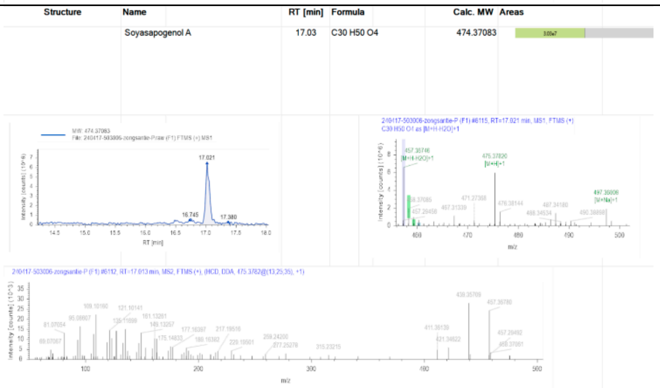
</td></tr>
<tr><td>Lupenone</td><td>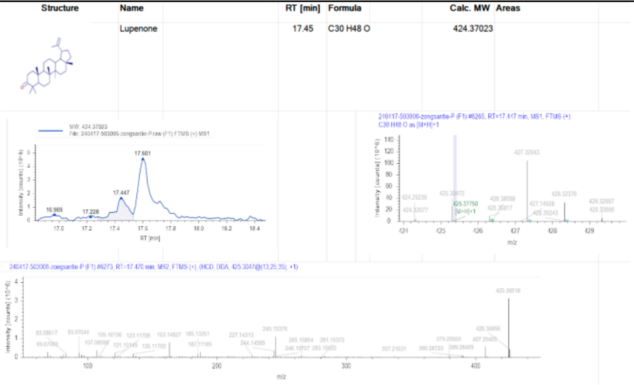
</td></tr>
</table>
